# Supplementary material for: Novel Antibiotics for Gram-Negative Nosocomial Pneumonia
Source: Antibiotics (Basel). 2024 Jul 5;13(7):629. doi: 10.3390/antibiotics13070629 (PMC11273951; doi:10.3390/antibiotics13070629)
Supplement: Supplementary file 1 [file antibiotics-13-00629-s001.zip › Supplementary Figure S1.pdf]

Supplementary material- Figure S1

Supplementary Figure S1. In vitro antimicrobial spectrum for newer antibiotics used for nosocomial pneumonia. *Each colour is based on current reported in vitro antibiotic susceptibility rates; these rates are indicative and may differ in different reports depending on cohorts and cut-offs used (EUCAST versus CLSI), and as new real-world in vitro sensitivity data arise constantly.*

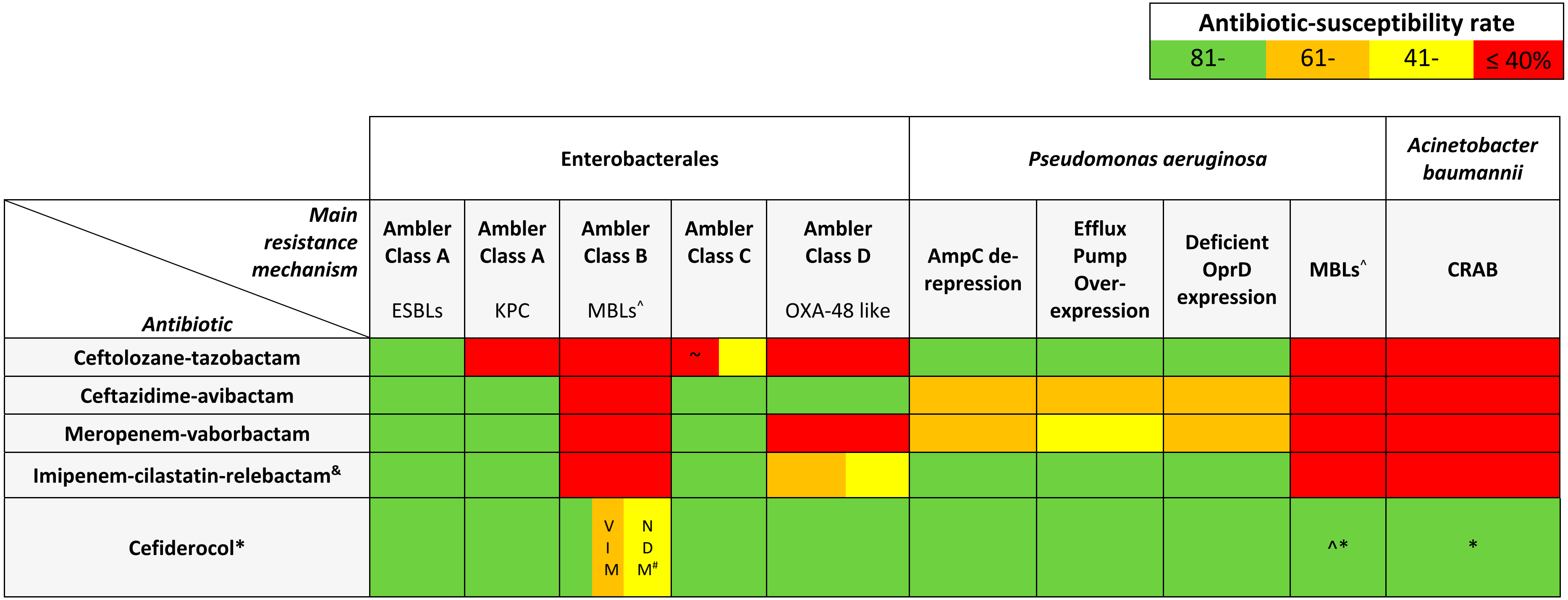

**Sulbactam-durlobactam**									

ESBL: extended-spectrum β-lactamase (e.g. **SHV**: sulphydral variable β-lactamase; **TEM**: named from the parental enzyme found in the blood of a Greek patient called Temoniera; **CTX-M**: cefotaxime-β-lactamases); **MBL**: metallo-b-lactamase (e.g. **VIM**: Verona-integron-mediated metallo-β-lactamase; **IMP**: imipenemase-type carbapenemase; **NDM**: New Delhi metallo-β-lactamase); **CRAB**: Carbapenem-Resistant *Acinetobacter baumannii.*

[&]The activity against OXA-48-producing strains is mostly that of imipenem; relebactam hardly enhances the activity of imipenem against OXA-48 (it has been reported to provide weak potentiation against some OXA-48 producing isolates of *Klebsiella pneumoniae*).

~ Ceftolozane developed to be more hydrolysis-resistant against Pseudomonas-derived AmpC compared to previous cephalosporins, however, much less is known about ceftolozane-tazobactam's activity against Enterobacterales-derived AmpC (tazobactam provides weaker protection against AmpC hydrolysis compared to newer beta-lactamase inhibitors) – in a study it has been reported that only 19% of *Enterobacter cloacae* were sensitive to ceftolozane-tazobactam.

[#]42–59% cefiderocol nonsusceptibility in NDM-producing clinical isolates reported in some cohorts.

^Enhanced stability does not always correlate with efficacy; *A combination of mechanisms, e.g. β-lactamases, mutations affecting expression or function of siderophore receptors and mutations affecting expression/function of porins or efflux pumps can increase cefiderocol strain resistance. Antibiotic heteroresistance is a phenotype in which a bacterial isolate contains subpopulations of cells showing a substantial reduction in antibiotic susceptibility compared with the main population. Cefiderocol heteroresistance was detected in carbapenem-resistant bacteria, including *Acinetobacter baumannii* (59%), *Klebsiella* spp (30%), *Pseudomonas aeruginosa* (9%), and *Stenotrophomonas maltophilia* (48%)*,* but its clinical importance remains unclear.

References

- Abniki R, Tashakor A, Masoudi M, Mansury D. Global Resistance of Imipenem/Relebactam against Gram-Negative Bacilli: Systematic Review and Meta-Analysis. Curr Ther Res Clin Exp. 2023 Oct 28;100:100723. doi: 10.1016/j.curtheres.2023.100723. PMID: 38174096; PMCID: PMC10758719.
- Andersson DI, Nicoloff H, Hjort K. Mechanisms and clinical relevance of bacterial heteroresistance. Nat Rev Microbiol. 2019 Aug;17(8):479-496. doi: 10.1038/s41579-019-0218-1. Epub 2019 Jun 24. PMID: 31235888.
- Barbier F, Hraiech S, Kernéis S, Veluppillai N, Pajot O, Poissy J, Roux D, Zahar JR; French Intensive Care Society. Rationale and evidence for the use of new beta-lactam/beta-lactamase inhibitor combinations and cefiderocol in critically ill patients. Ann Intensive Care. 2023 Jul 18;13(1):65. doi: 10.1186/s13613-023-01153-6. PMID: 37462830; PMCID: PMC10354316.
- Candel FJ, Santerre Henriksen A, Longshaw C, Yamano Y, Oliver A. In vitro activity of the novel siderophore cephalosporin, cefiderocol, in Gram-negative pathogens in Europe by site of infection. Clin Microbiol Infect. 2022 Mar;28(3):447.e1-447.e6. doi: 10.1016/j.cmi.2021.07.018. Epub 2021 Jul 21. PMID: 34298176.
- Castanheira M, Johnson MG, Yu B, Huntington JA, Carmelitano P, Bruno C, Rhee EG, Motyl M. Molecular Characterization of Baseline *Enterobacterales* and Pseudomonas aeruginosa Isolates from a Phase 3 Nosocomial Pneumonia (ASPECT-NP) Clinical Trial. Antimicrob Agents Chemother. 2021 Feb 17;65(3):e02461-20. doi: 10.1128/AAC.02461-20. PMID: 33318005; PMCID: PMC8092525.
- Choby JE, Ozturk T, Satola SW, Jacob JT, Weiss DS. Widespread cefiderocol heteroresistance in carbapenem-resistant Gram-negative pathogens. Lancet Infect Dis. 2021 May;21(5):597-598. doi: 10.1016/S1473-3099(21)00194-8. PMID: 33894839; PMCID: PMC8175093.

- Duda-Madej A, Viscardi S, Topola E. Meropenem/Vaborbactam: β-Lactam/β-Lactamase Inhibitor Combination, the Future in Eradicating Multidrug Resistance. Antibiotics (Basel). 2023 Nov 10;12(11):1612. doi: 10.3390/antibiotics12111612. PMID: 37998814; PMCID: PMC10668789.
- Dequin PF, Aubron C, Faure H, Garot D, Guillot M, Hamzaoui O, Lemiale V, Maizel J, Mootien JY, Osman D, Simon M, Thille AW, Vinsonneau C, Kuteifan K. The place of new antibiotics for Gram-negative bacterial infections in intensive care: report of a consensus conference. Ann Intensive Care. 2023 Jul 4;13(1):59. doi: 10.1186/s13613-023-01155-4. PMID: 37400647; PMCID: PMC10317910.
- Fraile-Ribot PA, Zamorano L, Orellana R, Del Barrio-Tofiño E, Sánchez-Diener I, Cortes-Lara S, López-Causapé C, Cabot G, Bou G, Martínez-Martínez L, Oliver A; GEMARA-SEIMC/REIPI Pseudomonas Study Group. Activity of Imipenem-Relebactam against a Large Collection of Pseudomonas aeruginosa Clinical Isolates and Isogenic β-Lactam-Resistant Mutants. Antimicrob Agents Chemother. 2020 Jan 27;64(2):e02165-19. doi: 10.1128/AAC.02165-19. PMID: 31740559; PMCID: PMC6985745.
- Granata G, Taglietti F, Schiavone F, Petrosillo N. Durlobactam in the Treatment of Multidrug-Resistant *Acinetobacter baumannii* Infections: A Systematic Review. J Clin Med. 2022 Jun 7;11(12):3258. doi: 10.3390/jcm11123258. PMID: 35743328; PMCID: PMC9225462.
- Iregui A, Khan Z, Landman D, Quale J. Activity of Cefiderocol Against *Enterobacterales*, *Pseudomonas aeruginosa*, and *Acinetobacter baumannii* Endemic to Medical Centres in New York City. Microb Drug Resist. 2020 Jul;26(7):722-726. doi: 10.1089/mdr.2019.0298. Epub 2020 Feb 7. PMID: 32031915; PMCID: PMC7368386.
- Kanj S, Bassetti M, Kiratisin P, Rodrigues R, Villegas MR, Yunsong Yu Y, van Duin D. Clinical data from studies involving novel antibiotics to treat multidrug-resistant Gram-negative bacterial infections. International Journal of Antimicrobial Agents. 2022; 60(3):106633. doi: 10.1016/j.ijantimicag.2022.106633. Epub 2022 Jul 1. PMID: 35787918.
- Karakonstantis S, Rousaki M, Kritsotakis EI. Cefiderocol: Systematic Review of Mechanisms of Resistance, Heteroresistance and In Vivo Emergence of Resistance. Antibiotics (Basel). 2022 May 27;11(6):723. doi: 10.3390/antibiotics11060723. PMID: 35740130; PMCID: PMC9220290.
- Karakonstantis S, Rousaki M, Vassilopoulou L, Kritsotakis EI. Global prevalence of cefiderocol non-susceptibility in Enterobacterales, Pseudomonas aeruginosa, Acinetobacter baumannii, and Stenotrophomonas maltophilia: a systematic review and meta-analysis. Clin Microbiol Infect. 2024 Feb;30(2):178-188. doi: 10.1016/j.cmi.2023.08.029. Epub 2023 Sep 4. PMID: 37666449.
- Livermore DM, Warner M, Mushtaq S. Activity of MK-7655 combined with imipenem against Enterobacteriaceae and Pseudomonas aeruginosa. J Antimicrob Chemother. 2013 Oct;68(10):2286-90. doi: 10.1093/jac/dkt178. Epub 2013 May 21. PMID: 23696619.
- Paul M, Carrara E, Retamar P, Tängdén T, Bitterman R, Bonomo RA, de Waele J, Daikos GL, Akova M, Harbarth S, Pulcini C, Garnacho-Montero J, Seme K, Tumbarello M, Lindemann PC, Gandra S, Yu Y, Bassetti M, Mouton JW, Tacconelli E, Rodríguez-Baño J. European Society of Clinical Microbiology and Infectious Diseases (ESCMID) guidelines for the treatment of infections caused by multidrug-resistant Gram-negative bacilli (endorsed by European society of intensive care medicine). Clin Microbiol Infect. 2022 Apr;28(4):521-547. doi: 10.1016/j.cmi.2021.11.025. Epub 2021 Dec 16. PMID: 34923128.
- Robin F, Auzou M, Bonnet R, et al. In vitro activity of ceftolozane-tazobactam against Enterobacter cloacae complex clinical isolates with different β-lactam resistance phenotypes. Antimicrob Agents Chemother 2018; 62:e00675-18.
- Shortridge D, Kantro V, Castanheira M. Meropenem-Vaborbactam Activity against U.S. Multidrug-Resistant *Enterobacterales* Strains, Including Carbapenem-Resistant Isolates. Microbiol Spectr. 2023 Feb 14;11(1):e0450722. doi: 10.1128/spectrum.04507-22. Epub 2023 Jan 9. PMID: 36622238; PMCID: PMC9927278.
- Viale P, Sandrock CE, Ramirez P, Rossolini GM, Lodise TP. Treatment of critically ill patients with cefiderocol for infections caused by multidrug-resistant pathogens: review of the evidence. Ann Intensive Care. 2023 Jun 15;13(1):52. doi: 10.1186/s13613-023-01146-5. PMID: 37322293; PMCID: PMC10272070.
- Wi YM, Greenwood-Quaintance KE, Schuetz AN, Ko KS, Peck KR, Song JH, Patel R. Activity of Ceftolozane-Tazobactam against Carbapenem-Resistant, Non-Carbapenemase-Producing Pseudomonas aeruginosa and Associated Resistance Mechanisms. Antimicrob Agents Chemother. 2017 Dec 21;62(1):e01970-17. doi: 10.1128/AAC.01970-17. PMID: 29133568; PMCID: PMC5740377.
