# Supplementary material for: Novel Antibiotics for Gram-Negative Nosocomial Pneumonia
Source: Antibiotics (Basel). 2024 Jul 5;13(7):629. doi: 10.3390/antibiotics13070629 (PMC11273951; doi:10.3390/antibiotics13070629)
Supplement: Supplementary file 1 [file antibiotics-13-00629-s001.zip › Supplementary Table S1.pdf]

Supplementary material-Table S1

Supplementary Table S1a. Dosing of novel antibiotics for nosocomial pneumonia.

Antibiotic	Infusion time	Standard dose[˜]	Dose in ARC[*] and renal impairment
**Ceftolozane-tazobactam**[&1]	1 hour (h)	3g every 8 h	ARC[*]: 3g every 8 h[2]<br><br>30≤GFR[*]<50 1.5 g every 8 h<br>15≤GFR[*]<30 0.75g every 8 h<br>15<GFR[*] 0.450g every 8 h<br>IHD* a single initial dose of 2.25 g should be followed by 0.450 g every 8 h
**Ceftazidime-avibactam**[3]	2 hours	2g/0.5g every 8 h	ARC[*] 2g/0.5g every 8 h[4]<br><br>30≤GFR[*]<50 1g/0.25g every 8 h<br>15≤GFR[*]<30 0.750g/0.1875g every 12 h<br>6≤GFR[*]<15 0.750g/0.1875g every 24 h<br>GFR[*]<6 or IHD[*] every 48 h or after dialysis session
**Meropenem-vaborbactam**[5]	3 hours	2g/2g every 8 h	ARC[*]: insufficient data regarding dosing-continuous infusion may be required.<br><br>20≤GFR[*]<40 1g/1g every 8<br>10≤GFR[*]<201g/1g every 12 h<br>GFR[*]<10 or IHD[*] 0.5g/0.5g every 12 h
**Imipenem-cilastatin-relebactam**[6]	30 minutes	500/500/250 mg every 6 h	ARC[*] 500/500 /250 mg every 6 h[7]<br><br>60≤GFR[*]<90 400/400/200 mg every 6 h<br>30≤GFR[*]<60 300/300/150 mg every 6 h<br>15≤GFR[*]<30 or IHD[*] 200/200/100 mg every 6 h<br><br>*For patients with GFR<15 imipenem-cilastatin-relebactam is not recommended unless IHD is commenced within 48 h from drug initiation.*
**Cefiderocol**[8]	3 hours	2g every 8 h	GFR≥120 2g every 6 h<br>30≤GFR[*]<60 1.5 g every 8 h<br>15≤GFR[*]<30 1g every 8 h<br>GFR[*]<15 or IHD[*] 0.75g every 12 h

**Sulbactam - durlobactam[9]**	3hours	1g/1g every 6 hours	GFR*≥130 1g/1g every 4 h<br>30≤GFR*<45 1g/1g every 8 h<br>15≤GFR*<30 1g/1g every 12 h<br>GFR*<15 or IHD* 1g/1g at 0 12 and 24 hours and then every 24 h

& Dose provided is for HAP/VAP- the dose for complicated intraabdominal and urinary infections is 50% of the above mentioned doses; *ARC, augmented renal clearance; GFR: Glomerular filtration rate, measured in ml/min; h: hour(s); IHD, intermitted hemodialysis, dose must be administrated after dialysis session is completed; ~For the first 24 to 48 hours, physicians should consider prescribing this dose, irrespective of patients' renal function. Renal impairment dose refers to the dose prescribed after the first 24-48 hours and is based on the calculated GFR in patients with stable renal function. In patients with Acute Kidney Injury, estimated GFR is a poor marker to assess renal function and dosing should be guided by monitoring antibiotic levels; # limited available data.

The tables are indicative- for prescribing, we refer the reader to the national and/or local guidelines.

Supplementary Table S1b. Dosing of novel antibiotics for nosocomial pneumonia during Continuous Renal Replacement Therapy (CRRT).

**Drug**	**Loading dose**	**Maintenance dose**			
		**Effluent rate**			
		<2 L/h	2L/h	3-4 L/h	>4L
**Ceftolazone-tazobactam[4,10,11]**	3 g	0.750 g every 8 h (h)	1.5g every 8 h	3g every 8 h	3g every 8 h
**Ceftazidime-avibactam~[12,13]**	2.5g	1.25-2.5 g every 8 h	2.5g every 8 h	IE*	IE*
**Cefiderocol[14]**	2g	1.5 g every 12 h	2g every 12 h	1.5 g every 8 h	2g every 8 h
**Imipenem-cilastatin-relebactam+[4,15]**	200 mg/100 mg every 6 hours (based on ex-vivo models)				
**Meropenem-vaborbactam$[4,16]**	1000 mg/1000 mg every 8 hours (data available from 1 case report only)				
**Sulbactam-durlobactam**	No available data				

~Evidence is limited and based on effluent rates of 2.5 L/h. The 2.5 g dose is recommended if resistant strain or difficult to treat infection;*IE: insufficient evidence

+No published PK data in critically ill patients with AKI or receiving CRRT. In ex vivo continuous hemofiltration (CH) and continuous haemodialysis (CHD) models, imipenem-cilastatin-relebactam (I-C-T) clearance was assessed and was shown that I-C-R was not removed by adsorption, but crossed effectively the hemodiafiltration membrane and a dose of 200 mg/100 mg every 6 hours was sufficient to achieve the

PK/PD targets. Note: although a warning regarding insufficient drug concentrations risk in cases of creatinine clearance>150 mL/min is found on I-C-T product specification, no dose adjustments are provided.

[%]Data only from a case report that PK characteristics of meropenem–vaborbactam (M-V) were assessed on an anuric patient on CRRT: 1000/1000 mg or even lower doses given as 3-hour infusion, achieved the PK/PD target for the isolated strain.

The tables are indicative- for prescribing, we refer the reader to the national and/or local guidelines.

References

1. HIGHLIGHTS of PRESCRIBING INFORMATION. https://www.merck.com/product/usa/pi_circulars/z/zerbaxa/zerbaxa_pi.pdf.

2. Bakdach, D.; Elajez, R.; Bakdach, A. R.; Awaisu, A.; De Pascale, G.; Ait Hssain, A. Pharmacokinetics, Pharmacodynamics, and Dosing Considerations of Novel β-Lactams and β-Lactam/β-Lactamase Inhibitors in Critically Ill Adult Patients: Focus on Obesity, Augmented Renal Clearance, Renal Replacement Therapies, and Extracorporeal Membrane Oxygenation. Journal of Clinical Medicine 2022, 11 (23), 6898. https://doi.org/10.3390/jcm11236898.

3. https://www.ema.europa.eu/en/documents/product-information/zavicefta-epar-product-information_en.pdf

4. Gorham, J.; Taccone, F. S.; Hites, M. Drug Regimens of Novel Antibiotics in Critically Ill Patients with Varying Renal Functions: A Rapid Review. Antibiotics 2022, 11 (5), 546. https://doi.org/10.3390/antibiotics11050546.

5.Vaborem 1 g/1 g powder for concentrate for solution for infusion - Summary of Product Characteristics (SmPC) - print friendly - (emc). www.medicines.org.uk. https://www.medicines.org.uk/emc/product/10813/smpc/print (accessed 2024-05-27).

6. HIGHLIGHTS of PRESCRIBING INFORMATION. https://www.merck.com/product/usa/pi_circulars/r/recarbrio/recarbrio_pi.pdf.

7. Fratoni, A. J.; Mah, J. W.; Nicolau, D. P.; Kuti, J. L. Imipenem/Cilastatin/Relebactam Pharmacokinetics in Critically Ill Patients with Augmented Renal Clearance. Journal of Antimicrobial Chemotherapy 2022, 77 (11), 2992–2999. https://doi.org/10.1093/jac/dkac261.

8. Fetcroja 1 g powder - Summary of Product Characteristics (SmPC) - print friendly - (emc). www.medicines.org.uk. https://www.medicines.org.uk/emc/product/11771/smpc/print (accessed 2024-05-27).

9. HIGHLIGHTS of PRESCRIBING INFORMATION. https://www.accessdata.fda.gov/drugsatfda_docs/label/2023/216974Orig1s000Correctedlbl.pdf.

10. Sime, F. B.; Lassig-Smith, M.; Starr, T.; Stuart, J.; Pandey, S.; Parker, S. L.; Wallis, S. C.; Lipman, J.; Roberts, J. A. A Population Pharmacokinetic Model-Guided Evaluation of Ceftolozane-Tazobactam Dosing in Critically Ill Patients Undergoing Continuous Venovenous Hemodiafiltration. Antimicrobial Agents and Chemotherapy 2019, 64 (1). https://doi.org/10.1128/aac.01655-19.

11. Aguilar, G.; Ferriols, R.; Martínez-Castro, S.; Ezquer, C.; Pastor, E.; Carbonell, J. A.; Alós, M.; Navarro, D. Optimizing Ceftolozane-Tazobactam Dosage in Critically Ill Patients during Continuous Venovenous Hemodiafiltration. Critical Care 2019, 23 (1). https://doi.org/10.1186/s13054-019-2434-5.

12. Gatti, M.; Rinaldi, M.; Paolo Gaibani; Siniscalchi, A.; Tonetti, T.; Giannella, M.; Viale, P.; Pea, F. A Descriptive Pharmacokinetic/Pharmacodynamic Analysis of Continuous Infusion Ceftazidime-Avibactam for Treating DTR Gram-Negative Infections in a Case Series of Critically Ill Patients Undergoing Continuous Veno-Venous Haemodiafiltration (CVVHDF). Journal of Critical Care 2023, 76, 154301–154301. https://doi.org/10.1016/j.jcrc.2023.154301.

13. Zhang, X.-S.; Wang, Y.-Z.; Shi, D.-W.; Xu, F.-M.; Yu, J.-H.; Chen, J.; Lin, G.-Y.; Zhang, C.-H.; Yu, X.-B.; Tang, C.-R. Efficacy and Pharmacodynamic Target Attainment for Ceftazidime–Avibactam Off-Label Dose Regimens in Patients with Continuous or Intermittent Venovenous Hemodialysis: Two Case Reports. Infectious diseases and therapy 2022, 11 (6), 2311–2319. https://doi.org/10.1007/s40121-022-00621-z.

14. Wenzler E, Butler D, Tan X, Katsube T, Wajima T. Pharmacokinetics, Pharmacodynamics, and Dose Optimization of Cefiderocol during Continuous Renal Replacement Therapy. Clin Pharmacokinet. 2022 Apr;61(4):539-552. doi: 10.1007/s40262-021-01086-y. Epub 2021 Nov 18. Erratum in: Clin Pharmacokinet. 2022 Jul;61(7):1069. PMID: 34792787; PMCID: PMC9167810.

15. Soo Min Jang; Yessayan, L.; Dean, M.; Costello, G.; Ravi Katwaru; Mueller, B. A. Imipenem/Relebactam Ex Vivo Clearance during Continuous Renal Replacement Therapy. Antibiotics 2021, 10 (10), 1184–1184. https://doi.org/10.3390/antibiotics10101184.

16. Kufel, W. D.; Eranki, A. P.; Paolino, K. M.; Call, A.; Miller, C. D.; Mogle, B. T. In Vivo Pharmacokinetic Analysis of Meropenem/Vaborbactam during Continuous Venovenous Haemodialysis. The journal of antimicrobial chemotherapy/Journal of antimicrobial chemotherapy 2019, 74 (7), 2117–2118. https://doi.org/10.1093/jac/dkz103.
