# Supplementary material for: Novel Antibiotics for Gram-Negative Nosocomial Pneumonia
Source: Antibiotics (Basel). 2024 Jul 5;13(7):629. doi: 10.3390/antibiotics13070629 (PMC11273951; doi:10.3390/antibiotics13070629)
Supplement: Supplementary file 1 [file antibiotics-13-00629-s001.zip › Supplementary Table S2.pdf]

Supplementary material – Table S2

Supplementary Table S2. EUCAST and CLSI MIC breakpoints for novel antibiotics

	**EUCAST MIC breakpoints (mg/L)**		**CLSI MIC Breakpoints (mg/L)**		
	Enterobacterales				
	S ≤	R >	S ≤	I	R ≥
CEFTO-TAZ	2*	2*	2/4	4/4	8/4
CEFTA	1	4	4	8	16
CEF-AVI	8~	8~	8/4[ξ]		16/4[ξ]
IMI (*Enterobacterales* except *Morganellaceae*)	2	4	1	2	4
IMI-REL (*Enterobacterales* except *Morganellaceae*)	2†	2†	1/4	2/4	4/4
MERO (indications other than meningitis)	2	8	1	2	4
MERO-VAB	8 ‖	8 ‖	4/8	8/8	16/8
CEF	2°	2 °	4 #	8 #	16 #
	***Pseudomonas* spp. º**				
CEFTO-TAZ (*P. aeruginosa*)	4*	4*	4/4	8/4	16/4
CEFTA	0.001	8	8	16	32
CEF-AVI (*P. aeruginosa*)	8~	8~	8/4		16/4
IMI	0.001	4	2	4	8
IMI-REL (*P. aeruginosa*)	2†	2†	2/4	4/4	8/4
MERO (indications other than meningitis)	2	8	2	4	8
MERO-VAB (*P. aeruginosa*)	8 ‖	8 ‖	No AST performed		
CEF (*P. aeruginosa*)	2°	2°	4 #	8 #	16 #
	***Acinetobacter* spp**				
CEFTO-TAZ	-	-	No AST performed		
CEFTA	-	-	8	16	32
CEF-AVI	-	-	No AST performed		
IMI	2	4	2	4	8
IMI-REL	Note A	Note A	No AST performed		
MERO (indications other than meningitis)	2	8	2	4	8
MERO-VAB	Note A	Note A	No AST performed		
CEF (*Acinetobacter Baumannii* complex)	IE	IE	4 #	8 #	16 #
SUL-DUR	No EUCAST breakpoints defined		4/4	8/4	16/4
	Stenotrophomonas maltophilia				
CEF	IE	IE	1 #		

Abbreviations: AST, Antimicrobial Susceptibility Testing; CEF, cefiderocol; CEF-AVI, ceftazidime-avibactam; CEFTA, Ceftazidime; CEFTO-TAZ, ceftolozane-tazobactam; CLSI, Clinical & Laboratory Standards Institute; EUCAST, European Committee on Antimicrobial Susceptibility Testing; IMI, imipenem; IMI/REL, imipenem-cilastatin/relebactam; MERO, meropenem; MERO-VAB, meropenem-vaborbactam; MIC, minimum inhibitory concentration; SUL-DUR, sulbactam– durlobactam

- : The agent is unsuitable for treatment. Susceptibility testing is not recommended
IE: Insufficient evidence that the organism or group is a good target for therapy with the agent

* For susceptibility testing purposes, the concentration of tazobactam is fixed at 4 mg/L.

~For susceptibility testing purposes, the concentration of avibactam is fixed at 4 mg/L.

ξ Confirmatory MIC testing is indicated for isolates with zones of 20–22 mm to avoid reporting false-susceptible or false-resistant results.

† For susceptibility testing purposes, the concentration of relebactam is fixed at 4 mg/L.

‖ For susceptibility testing purposes, the concentration of vaborbactam is fixed at 8 mg/L

° Broth microdilution MIC determination must be performed in iron-depleted Mueller-Hinton broth and specific reading instructions must be followed.

The accuracy and reproducibility of cefiderocol testing results by disk diffusion and broth microdilution are markedly affected by iron concentration and inoculum preparation and may vary by disk and media manufacturer. Depending on the type of variance observed, false-resistant or false-susceptible results may occur. Testing subsequent isolates is encouraged. Discussion with prescribers and antimicrobial stewardship members regarding the potential for inaccuracies is recommended.

º CLSI breakpoints apply for *P. aeruginosa*.

Note A: The beta-lactamases produced by the organisms either do not modify the parent carbapenem or are insufficiently inhibited by the inhibitor. Therefore the addition of the beta-lactamase inhibitor does not add clinical benefit.

References:

1. EUCAST Version 14.0, valid from 2024-01-01. https://www.eucast.org/fileadmin/src/media/PDFs/EUCAST_files/Breakpoint_tables/v_14.0_Breakpoint_Tables.pdf, accessed on 26 June 2024

2. CLSI M100-ED34:2024 Performance Standards for Antimicrobial Susceptibility Testing, 34th Edition. https://em100.edaptivedocs.net/GetDoc.aspx?doc=CLSI%20M100%20ED34:2024&xormat=SPDF&src=BB, accessed on 26 June 2024
